# Recombination events drives the emergence of Colombian *Helicobacter pylori* subpopulations with self-identity ancestry

**DOI:** 10.1080/21505594.2022.2095737

**Published:** 2022-07-15

**Authors:** Alix A. Guevara-Tique, Roberto C. Torres, Maria M. Bravo, Luis G. Carvajal Carmona, María M. Echeverry de Polanco, Mabel E. Bohórquez, Javier Torres

**Affiliations:** a Grupo de Investigación en Citogenética, Filogenia y Evolución de Poblaciones, Departamento de Ciencias y Ciencias de la Salud, Universidad del Tolima, Tolima, Colombia; b The Center for Microbes, Development and Health, CAS Key Laboratory of Molecular Virology and Immunology, Institut Pasteur of Shanghai, Chinese Academy of Sciences, Shanghai, China; c c Unidad de Investigación en Enfermedades Infecciosas, UMAE Pediatria, Instituto Mexicano del Seguro Social, México; d Laboratorio de Inmunología, Instituto Nacional de Cancerología, Bogotá, D. C., Colombia; e Genome Center, Department of Biochemistry and Molecular Medicine, School of Medicine-University of California, Davis, California, USA; f Medicine Program, Department of Health Sciences, Tolima University, Tolima, Colombia

**Keywords:** *Helicobacter pylori*, Colombia, population structure, ancestry, evolution, recombination

## Abstract

*Helicobacter pylori* have coevolved with mankind since its origins, adapting to different human groups. In America, *H. pylori* has evolved into several subpopulations. We analysed the genome of 154 Colombian strains along with 1,091 strains from worldwide populations to discern the ancestry and adaption to Colombian people. Population structure and ancestry was inferred with FineStructure and ChromoPainter. Phylogenetic relationship and the relative effect of recombination were analysing the core SNPs. Also, a Fst index was calculated to identify the gene variants with the strongest fixation in the Colombian subpopulations compared to their parent population *hspSWEurope*. FineStructure allowed the identification of two Colombian subpopulations, the previously described *hspSWEuropeColombia* and a novel subpopulation named *hspColombia*, that included three subgroups following their geographic origin. Colombian subpopulations represent an admixture of European, African and Indigenous ancestry; although some genomes showed a high proportion of self identity, suggesting an advanced adaption to these mestizo Colombian groups. We found that recombination is more important that punctual mutations in *H. pylori* genome diversity, 13.9 more important in *hspSWEurope*, 12.5 in *hspSWEColombia* and 10.5 in *hspColombia*, reflecting the divergence of these subpopulations. Fst analysis identified 82 SNPs fixed in 26 genes of the *hspColombia* subpopulation that encode for outer membrane and central metabolism proteins. Strongest fixation indexes were identified in genes encoding HofC, HopE, FrpB-4 and Sialidase A. These findings demonstrate that *H. pylori* has evolved in Colombia to give rise to subpopulations with a self identity ancestry, reflected in allele changes on genes encoding for outer membrane proteins.

## Introduction

The association of *H. pylori* with humans dates back at least 58,000 y, most probably since the origin of our species and accompanied humans during its migrations out of Africa [1–3]. Genome analyzes estimates that East Asians populated the Americas about 23 kya and dispersed across the continent after a settlement in Beringia 8,000 y ago [4]. Thus, the first Indigenous Americans carried bacterial strains with Asian ancestry, which later evolved in isolation through a process of bacterial recombination and/or mutation. However, genome diversity of *H. pylori* in the Americas showed a drastic change after the arrival of Europeans and the transatlantic trade of African slaves starting 500 y ago, leading to genomes with a mosaic of admixed ancestries that have followed unique evolutionary paths [5–7].

The Colombian population has a complex and diverse genetic structure as a result of migrations of different human groups separated by origin, time, and space [8,9]. Cultural, geographical, and climatic characteristics delimit the country in six regions, Andean, Pacific, Caribbean, Orinoquia, Amazonian and Insular. The human groups with African ancestry are concentrated in the Caribbean and Pacific regions, whereas the Indigenous people with Amerindian ancestry are found in the Amazon. People from the Andean region are mostly mestizo but with a high European ancestry component [8]. Initial studies of *H. pylori* demonstrated that the mountain regions of the country have the highest incidence and mortality of gastric cancer (GC) associated with the presence of *H. pylori* strains with European ancestry, while in the coast regions a low risk of GC has been reported that correlated with a high prevalence of African strains (*hpAfrica*) [10–14]. More recent studies have revealed the presence of clearly differentiated Colombian *H. pylori* groups although with a defined European ancestry [7,15–17]. In order to better understand the population structure and admixture of *H. pylori* in Colombia, we analysed the complete genome of 154 Colombian strains together with 1,091 publicly available genomes that represent the already described worldwide subpopulations. In addition, we aimed to identify the main evolutionary factors that contribute to the divergence of *H. pylori* and the gene variants that distinguished the Colombian strains from their parent population *hspSWEurope.*

## Materials and methods

### Pylorigenomes studied

A total of 157 Colombian *H. pylori* genomes publicly available at Enterobase and Genbank (NCBI) were included in this study, corresponding to strains isolated from patients residing in 10 departments of the country (figure S1). In addition, 1,102 *H. pylori* genomes from 46 countries were included to represent the different populations of this bacterium reported worldwide; 82 of these assemblies were obtained from previous works [16–18], while the 1020 remaining *H. pylori* were downloaded from Enterobase, Genbank and BiGS databases (table S1, figure S1 and S2). The 1,259 genomes were annotated with Prokka v1.12[19] and then filtered according to genomic features, discarding 14 strains with a genome length greater than 1.84 Mbp, with more than 474 contigs or a gene content greater than 1,696 genes (figure S3). The final number of selected samples was 1,245, including 154 strains from Colombia and 1,091 from other parts of the world (table S1).

## Population structure of H. pylori in Colombia

Genome sequences of included samples were mapped against the genome of the *H. pylori* 26,695 reference strain (NC_000915.1) to call the SNPs from the coregenome (core – SNPs) as implemented by the Snippy pipeline [20] using the – contig option and with a subsequent filter of those variants with a minimum allele frequency greater than 5%. The population structure was inferred from the obtained 195,217 core – SNPs using FineSTRUCTURE v2 and ChromoPainter v2[21]as described by Yahara et al., 2013 [22]. Population assignment of each sample was based on the observed clusters in the co – ancestry matrix using as reference the population labels of 938 samples reported in previous works [7,7,15–17]. To infer the ancestry proportions and identify admixture in Colombian *H. pylori* strains we conducted a “chromosome painting” using ChromoPainter v2 [21,] designating a collection of 1,245 Colombian and non – Colombian genomes as donors to paint each of 154 Colombian recipient genomes.

## Phylogenetic reconstruction

To explore the phylogenetic history of *H. pylori* we first made a phylogeny based on the SNPs from the coregenome, analysing the entire dataset of 1,245 genomes in MEGA X v10.2.6 [23] using a Neighbour – Joining method. Then, to investigate about the factors that drive the divergence between *H. pylori* populations we generated a phylogenetic tree based on genetic distances between all subpopulations using a maximum likelihood (ML) method with FastTree v2 [24]. All the phylogenetic trees were visualized with iTOL v4 [25].

## Genetic diversity in Colombian subpopulations

To better understand the divergence between *H. pylori* subpopulations, we first performed a principal components analysis with the prcomp R package, and compared the average distances between groups, using as input the haplotype data from the coregenome obtained for the 1,245 strains. After this general overview of the divergence, we aimed to compare the admixture degree of the strains and the weight that recombination events have had against the weight generated by punctual mutations in the divergence of the different populations. To this end, we analysed all the samples available for *hspColombia* and *hspSWEuropeColombia* and a subset of 54 *hspSWEurope* genomes from Spain, Portugal, France and Belgium to include strains from countries involved in the conquest of the Americas. In addition, we also analysed 20 representative strains of each *H. pylori* worldwide subpopulations, to include the 10 most admixed and the 10 less admixed (purest) strains of each group according to the ChromoPainter outputs. The ratio of the recombination to mutation (R/θ) was estimated using the tree generated with FastTree v2 [24]. as input in ClonalFrameML [26]. Finally, the per – branch relative effect of recombination to mutation (r/m) was estimated running ClonalFrameML with the – embranch option and the initial values for R/θ = 0. 680,231, the inverse mean of the DNA import length (1/δ) = 0.0080466, and the mean divergence of imported DNA (nu) = 0.0552114, calculated with the standard model of ClonalFrameML.

## Gene variants fixed in hspColombia subpopulation

To identify genetic variants that characterize the *hspColombia* subpopulation we analysed changes in allele frequency between strains from the *hspColombia* subpopulation and its ancestral population *hspSWEurope*. Although we noted a trend toward stabilization of the number of SNPs in the coregenome of each subpopulation when we include 16 strains or more, we decided to exclude the *hspSWEuropeColombia* subpopulation from this analysis due to the low number of strains of this population (n = 22 strains).

First, we estimated the coregenome and extracted the SNPs as described above, considering all the samples available for *hspColombia* and 54 strains from *hspSWEuropeColombia*, then we used the SNPs as input to compute a fixation index (Fst) as implemented in VCFtools [27]. Fst values of each position in the coregenome were visualized in R v4.0.5 [28]. The sequences of 26 coregenes with a Fst values >0.5 were extracted and aligned by subpopulation using the Muscle software [29]. Then, we evaluated the confidence of selected positions and kept only those with *P* values <0.05 (Table 1). Consensus nucleotide and protein sequences of these 26 genes were then aligned to the *H. pylori* 26,695 reference strain and visualized with WebLog [30] to identify positions corresponding to synonymous and non – synonymous (NS) substitutions in each subpopulation. Finally, the structure of proteins with at least two NS mutations were obtained from the Protein Data Bank [31] when available or predicted in the I – TASSER server [32], and visualized in the UCSF Chimera v1.5 [33] program, using a gradient colour to highlight Fst values. In addition, to compare the effects of NS mutations in the predicted 3D structure of genes with highest Fst values, we analysed the consensus sequences of the 8 proteins with largest amount of NS mutations by each subpopulation.

**Table 1.** Genes with a high FST value a position between *hspColombia* and *hspSWEurope*.

Gene*	Description	Total of SNPs	Number of SNPs Fst >0.49	Number of SNPs with a Non-Synonymous effect	Number of codons in change	Fst max value	Location Protein	Biological Function
*HP0018*	Hypothetical protein	84	1	1	1	0. 678,227	Unkown	Survival
*HP0019*	Chemotaxis protein CheV	62	1	1	1	0. 66,627	Membrane	Survival
*HP0127*	Outer membrane protein HorB	56	2	2	1	0. 622,084	Membrane	Survival
*HP0130*	Hypothetical protein	33	1	1	1	0. 635,371	Unkown	Survival
*HP0175*	Putative peptidyl – prolyl cis – trans isomerase PpiC	40	2	0	0	0. 641,939	Cytoplasm	Central Metabolism
*HP0181*	Membrane protein required for colicin V production	45	1	0	0	0. 500,663	Membrane	Survival
*HP0252*	Outer membrane protein HopF	88	4	2	2	0. 635,204	Membrane	Survival
*HP0269*	(dimethylallyl) Adenosine tRNA methylthiotransferase	125	1	0	0	0. 504,857	Cytoplasm	Central Metabolism
*HP0377*	Thiol:disulphide interchange protein	38	4	3	2	0. 674,541	Membrane	Survival
*HP0415*	Potassium efflux system protein/Small – conductance mechanosensitive channel	110	1	1	1	0. 50,346	Membrane	Survival
*HP0486*	Outer membrane protein HofC	73	19	10	7	0. 937,532	Membrane	Survival
*HP0517*	GTP – binding protein Era	72	4	4	3	0. 609,185	Cytoplasm	Central Metabolism
*HP0554*	Sialidase A	34	6	5	3	0. 687,286	Membrane	Survival
*HP0558*	3-oxoacyl-(acyl carrier protein) synthase II	51	1	0	0	0. 506,588	Cytoplasm	Central Metabolism
*HP0564*	Uncharacterized protein	14	1	0	0	0. 625,088	Cytoplasm	Central Metabolism
*HP0686*	Iron(III) dicitrate transport protein FecA	69	2	1	1	0. 54,699	Membrane	Survival
*HP0706*	Outer membrane protein HopE	61	9	8	6	0. 86,328	Membrane	Survival
*HP0788*	Outer membrane protein HofF	70	1	1	1	0. 623,103	Membrane	Survival
*HP1012*	Putative zinc protease PqqE	99	1	1	1	0. 589,325	Membrane	Survival
*HP1054*	Hypothetical protein	66	1	1	1	0. 535,516	Membrane	Survival
*HP1055*	Hypothetical protein	24	6	3	3	0. 717,047	Membrane	Survival
*HP1056*	Hypothetical protein	23	1	0	0	0. 511,819	Membrane	Survival
*HP1340*	Biopolymer transport protein ExbD	23	1	1	1	0. 63,672	Membrane	Survival
*HP1487*	ABC-2 type transport system permease protein	53	3	2	2	0. 540,672	Membrane	Survival
*HP1512*	Iron – regulated outer membrane protein FrpB4	82	5	3	1	0. 799,812	Membrane	Survival
*HP1565*	Penicillin – binding protein 2	103	2	2	2	0. 584,235	Membrane	Survival

*Gene Id corresponds to the ORF in 26,695 strain.

## Results

### Population structure of of *H.* *pylori* and novel subpopulations in Colombia and Australia

**Figure 1.**
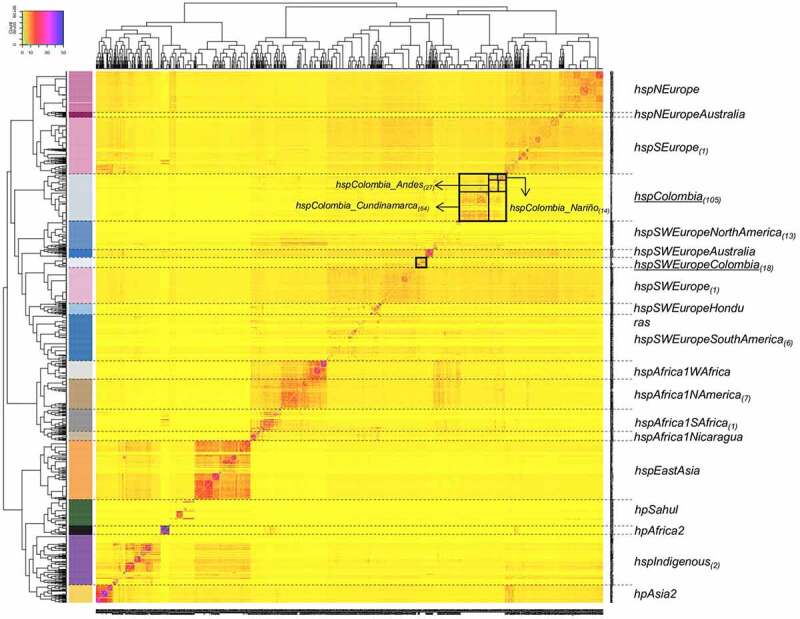
**Co – Ancestry matrix showing the population structure and genetic flow of the 1245 *H. pylori* strains analysed**. the colour gradient in the heat map corresponds to the number of genomic fragments imported from a donor genome (column) to a recipient genome (row). The inferred tree is displayed at the top and left of the heat map, and *H. pylori* strain names are at the bottom and right. The allocation of the *H. pylori* population is provided in colours on the left side of the heat map and the names of each population group are located on the right; the subscripts indicate the number of Colombian strains identified in each population. The black boxes indicate the two subpopulations identified and the geographic subclusters identified within *hspColombia*.

The analysis of the population structure with FineSTRUCTURE for the 1,245 strains allowed the identification of 19 populations and subpopulations (Figure 1). The genomes included in the analysis came from 46 countries, some of them poorly represented in previous studies, such as Australia and Papua New Guinea (table S1), which allowed the identification of new subpopulations (*hspNEuropeAustralia* and *hspSWEuropeAustralia*). The resulted co – ancestry matrix showed three main clusters, one mostly composed by Asian strains that includes the *hpAsia2, hpEAsia, hspIndigenous*, and *hpSahul*; *hpAfrica2* also felt within this large group. The second cluster was composed by African populations, including *hspAfrica1SAfrica*, *hspAfrica1Wafrica*, *hspAfrica1Namerica* and *hspAfrica1Nicaragua*. The third and biggest cluster was defined by populations with European ancestry, and it divided intwo subclusters, one composed by *hspSEurope*, *hspNEurope* and the novel subpopulation *hspNEuropeAustralia*, not described previously and that included exclusively *H. pylori* genomes from Australia. The second subcluster was composed by the *hspSWEurope*, *hspSWEuropeHonduras, hspSWEuropeColombia* and the novel Colombian cluster named *hspColombia*. Within this subcluster felt what we previously described as subpopulations *hspSWEuropeMexico* and *hspAfrica1MiscAmerica* [7,16]; but now reviewed after extending the study to include more strains from Latin America we renamed these two last groups as *hspSWEuropeNorthAmerica* and *hspSWEuropeSouthAmerica* (Figure 1). Furthermore, within this subcluster we also identified an Australian subpopulation not described before and that we named *hspSWEuropeAustralia* (Figure 1).

The large number of Colombian and worldwide strains allowed the identification of two clearly separated Colombian subpopulations (Figure 1, arrows), the previously reported as *hspSWEuropeColombia* (because of its closeness with *hspSWEurope* strains) by Muñoz et al., (2017) [16] and the one not previously described that we designated as *hspColombia*. This last population was further separated in three subclusters, which corresponded to their region of origin and were named as *hspColombia_Nariño* that included only isolates from the Nariño department; *hspColombia_Andes* composed mainly with strains from departments of the Andes Mountain region and *hspColombia_Cundinamarca* composed mostly by strains isolated in the Cundinamarca department (figure S4). The separation of these subgroups was also evidenced in a phylogenetic tree built only with the strains belonging to Colombian subpopulations (figure S5).

Most of the Colombian strains (80%) belonged to either *hspSWEuropeColombia* (18) or to *hspColombia* (105) and the remaining belonged to other 7 subpopulations, including *hspSWEuropeNorthAmerica* (13), *hspAfrica1Namerica* (7), *hspSWEuropeSouthAmerica* (6), *hspIndigenous* (2), *hspSWEurope* (1), *hspSEurop*e (1) and *hspAfrica1Safrica* (1) (Figure 1, table S2).

## Divergence of the *H.* *pylori* populations

**Figure 2.**
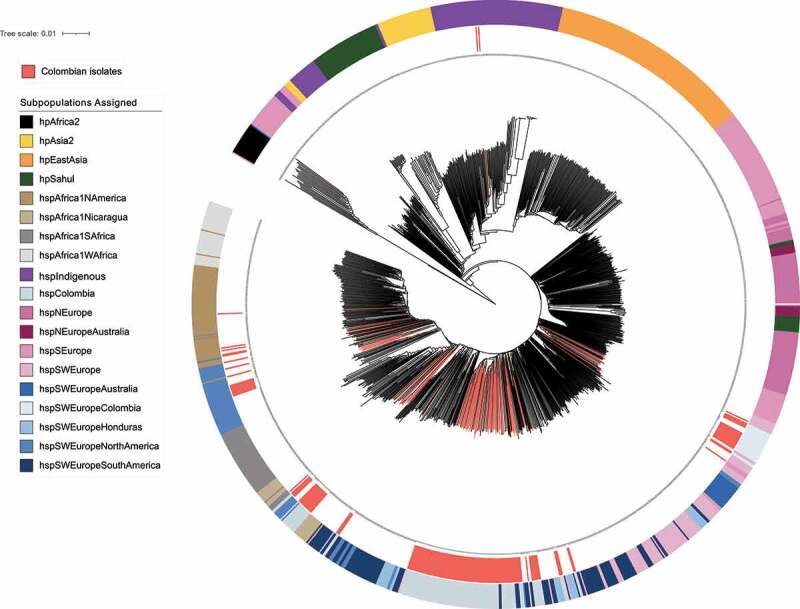
**Phylogenetic reconstruction of the 1245 *H. pylori* strains based the coregenome SNPs**. the phylogenetic tree was calculated using the neighbor – joining model in MEGA X v10.2.6 and it was edited in iTOL v4. The blue highlighted background indicates the group formed by the Colombian strains belonging to the *hspSWEuropeColombia* subpopulation (n = 18) and the coral shows the groups formed by Colombian strains belonging to the *hspColombia* (n = 105). The internal ring and coloured branches represent the 154 Colombian *H. pylori* isolates analysed; the colours of the outer ring represent the *H. pylori* subpopulations assigned in the population structure analysis. Non clear separation of European and Latin American subpopulations was observed.

The clonal genealogy relationship of *H. pylori* showed well – defined clades for the previously reported populations *hpAsia2*, *hpEastAsia*, *hpSahul, hspAfrica1Wafrica, hspAfrica1Safrica* and *hspAfrica1Namerica*. It was also evident that *hpAfrica2* was grouped far from all other populations, which agrees with Linz et al., (2007) [1], who suggested this group as one of the most ancient *H. pylori* lineages. Another big node of the tree was mostly composed by *hspSEurope* and *hspNEurope*, the latter including two external nodes of the new proposed *hspNEuropeAustralia* population, the next node was composed by populations derived from *hspSWEurope*, including clusters of strains from the *hspColombia p*opulation (Figure 2).

**Figure 3.**
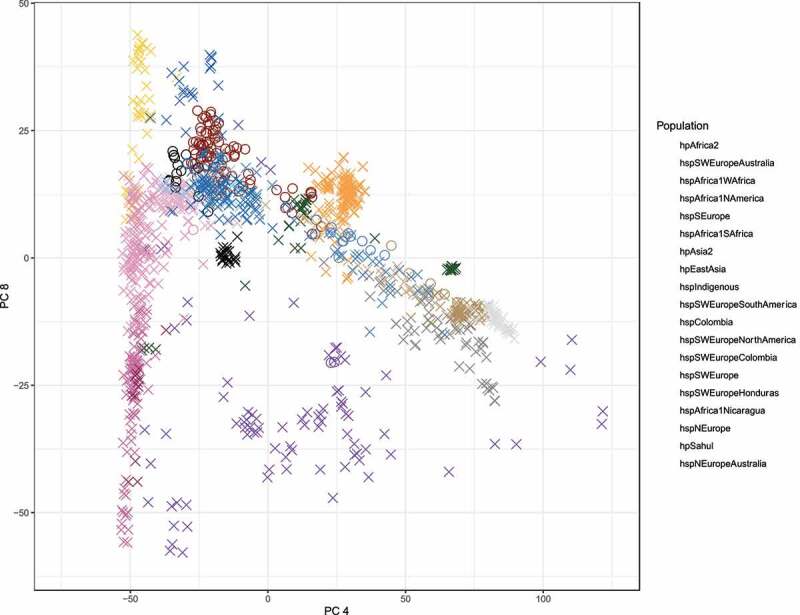
**Principal component analysis of 1,245 studied strains**. strains from Colombia are represented in circles; those with black ringes indicate the Colombian strains belonging to the *hspSWEuropeColombia* subpopulation (n = 18) and the red ring show the groups formed by Colombian strains belonging to the *hspColombia* (n = 105). Strains from all other populations are represented as X and the inner colours represent the *H. pylori* subpopulations assigned in the population structure analysis. .

**Figure 4.**
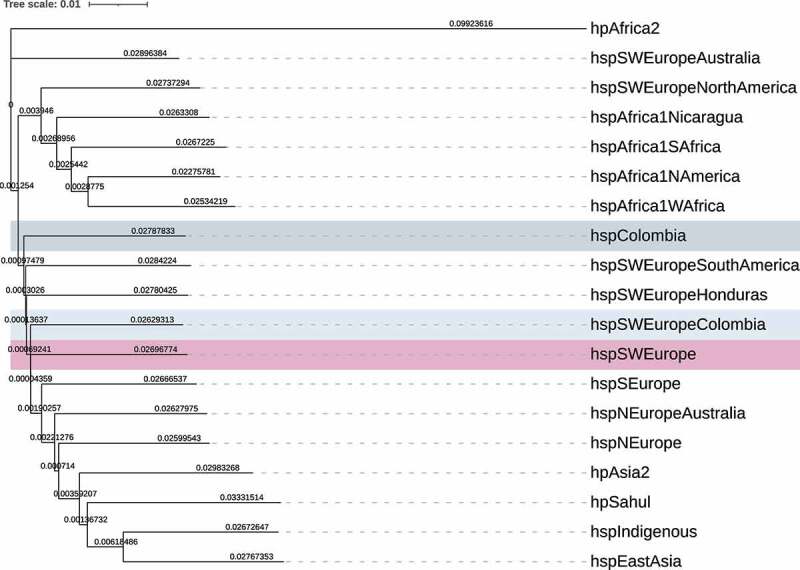
**Phylogenetic reconstruction based on the genetic distance between the subpopulations identified**. a clear separation of subpopulations identified in FineStructure was observed. The *hspColombia* subpopulation show a higher evolutionary divergence from *hspSWEurope*, compare with *hspSWEuropeColombia*, that is next to it. The light blue highlighted background indicates the *hspSWEuropeColombia* subpopulation (n = 22), grey correspond to *hspColombia* (n = 105), and the fuchsia shows the *hspSWEurope* (n = 85). The phylogenetic tree was calculated using FastTree v2.1.

In addition, to the phylogenetic reconstruction, we investigated the divergence of populations performing a principal component analysis (PCA). To better differentiate the Colombian subpopulations, we looked for the axis with the best separation of the populations with European ancestry. The clusters of the PCA agree with those observed in the coancestry matrix defining the populations boundaries better than the clonal genealogy (Figure 3). In the right most side of the PCA we observed a cluster of African strains, including *hspAfrica1Wafrica, hspAfrica1Safrica, hspAfrica1Namerica* and *hspAfrica1Nicaragua*, followed by *hpEasAsia* and a large mixed cluster composed by samples from Europe, America and Australia related with the *hspSWEurope* subpopulation, even when these subpopulations were very closed to each other, the boundaries were well defined. Finally, we observed a cluster composed by European subpopulations from the old world and Australia, including *hspNEurope, hspNEuropeAustraila* and *hspSEurope*. Of note, this axis also showed the clusters of *hpSahul, hpAsia2, hspIndigenous* and *hpAfrica2* as the most divergent subpopulations. This population structure was also clearly supported when we measure the average distance between populations (Figure 4). The largest average distance was observed between the *hspColombia* and the parental population *hspSWEurope* (0.0561), while the closer distance was between *hspSWEurope* and *hspSWEuropeColombia* (0.0533).

## Recombination as the main factor of divergence in H. pylori and Colombian subpopulations

To further investigate the evolutive factors that drives the divergence of *H. pylori* we estimated the relative effect of recombination to mutation between all strains in the “pure and mixed” subset (see methods) using ClonalFrameML. We first obtained a value of R/θ = 0. 680,231, 1/δ = 0.0080466, and nu = 0.0552114 running ClonalFrameML with the default parameters. Then, we used these values as initial parameters to run the per branch model of ClonalFrameML, obtaining the values R/θ = 1.3377, 1/δ = 0.0148164, nu = 0.0335464 and a relative effect of recombination to mutation (r/m) = 1.3, which indicate that recombination occurs three times more frequent and caused 1.3 times more substitutions than mutation in *H. pylori*. Then, we investigated this effect for *hspSWEurope*, *hspSWEuropeColombia* and *hspColombia* subpopulations, estimating that recombination occurs two times more often (R/θ = 1.9) and causes 14 times more substitutions (r/m = 13.9) than mutation in *hspSWEurope;* whereas in *hspColombia* (R/θ = 1.5, r/m = 12.5) and *hspSWEuropeColombia* (R/θ = 1.5, r/m = 10.5) recombination was also more frequent than mutation (Table 2). Of note, we also identified that the novel *hspNEuropeAustralia* has the highest relative effect of recombination to mutation (r/m = 17.59) while the lower effect was identified in *hspAfrica2* and *hspAfrica1WAfrica*, where mutations were more frequent than recombination, with values of r/m = 0.6 and r/m = 0.9, respectively (table S3).

**Table 2.** Relative effect of recombination to the mutation in Colombian and *hspSweurope* populations.

Parameter (avg)	*hspSWEurope*			*hspSWEuropeColombia*			*hspColombia*		
	All	High self– Identity	Mixed	All	High self – Identity	Mixed	All	High self – Identity	Mixed
Recombination/mutation rate (R/θ)	1.932	1.949	1.911	1.503	0.924	1.834	1.509	1.204	1.753
Length of imports (δ)	129.805	109.573	150.037	114.527	62.297	144.372	135.193	111.029	154.524
Divergence of imports between donors and receptors (v)	0.055	0.054	0.055	0.052	0.047	0.055	0.053	0.049	0.057
Number of substitutions per recombination	6.957	5.943	7.971	6.117	3.117	7.832	7.467	5.864	8.749
Relative effect of recombination to mutation (r/m)	13.914	11.600	16.227	10.554	3.503	14.584	12.504	9.184	15.160

## Admixture and diversity in Colombian subpopulations of *H.* *pylori*

**Figure 5.**
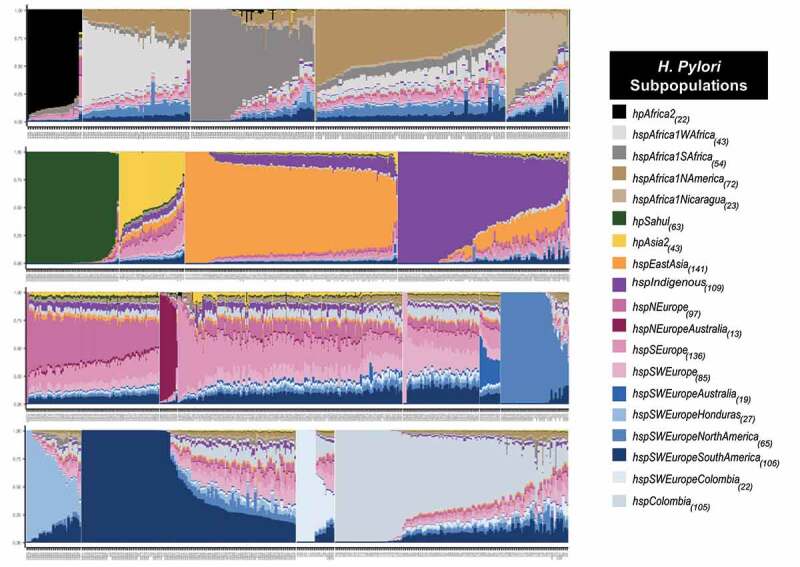
**Population admixture in *H. pylori* strains**. Samples were clustered and sorted according to the admixture degree in the coregenome. From left to right and from top to down, African, Asian, American, European, and Colombian subpopulations. Each column represents a strain, and the colour indicates the proportion of ancestry in that sample. The subscripts indicate the number of strains identified in each population.

A high proportion of the genomes of the old – world populations *hpAfrica2, hpEastAsia, hpSahul* and *hspNEuropeAustralia* are painted by their own population (self – identity). In contrast, most populations with European ancestry (*hspNEurope, hspSEurope*, and *hspSWEurope*) present high admixture, contributed by different populations (Figure 5). To investigate whether the recombination has an impact in the emergence of strains with self – identity ancestry, we compared the relative effect of recombination to mutation in 10 strains with the most self – identity against to 10 strains with the highest admixture in each of the populations that include a clear differentiation of the admixture degree. The results showed that on average, the strains with highest self – identity presented a lower relative effect of recombination to mutation compared to the highly admixed strains which showed a factor of 3 (*hspSWEuropeNorthAmerica*) to 13 (*hspIndigenous*) times more recombination, except for *hspSEurope* and *hpEastAsia* which the effect was higher in mixed strains (table S3). In the Colombian subpopulations, contribution from European, African, and Amerindian donors was observed, but self – identity was the predominant ancestral component in some of the strains (figure S6a). Here, the factor of the relative recombination effect to mutation between mixed and self – identity strains was 11 times more important in *hspSWEuropeColombia* strains, while it was 5 times higher in mixed than in self – identity strains for *hspColombia* (Table 2). Of note, several genomes of isolates from *hspColombia_Nariño, hsp Colombia_Andes*, and *hspColombia_Cundinamarca* were completely painted by their own subpopulations, showing almost 100% of self – identity (figure S6b).

## Genes coding for outer membrane proteins are fixed in the hspColombia subpopulation

**Figure 6.**
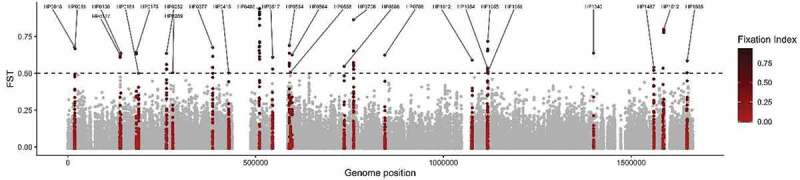
**Core – Genome Fst analysis to identify genetic variants that are significantly more common in the *hspColombia* than in its parental subpopulation *hspSWEurope***. the X – axis indicates the nucleotide position according to the 26,695 *H. pylori* reference genome and the Y – axis shows the Fst value for each site. In red was show the 26 genes that present a site with an FST value of 0.5 or greater.

**Figure 7.**
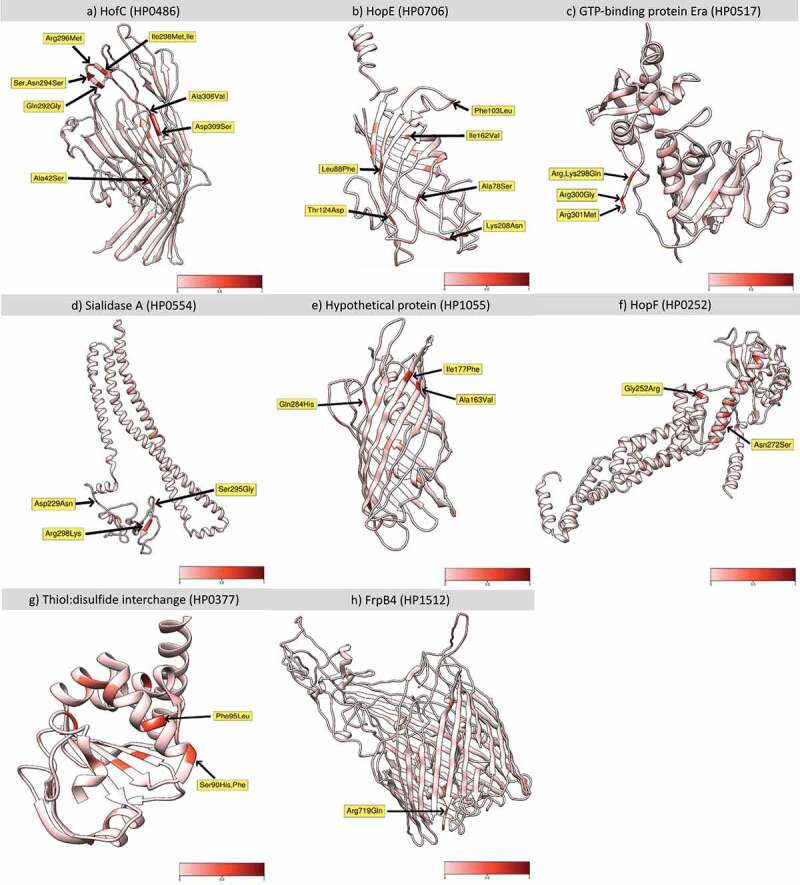
**Non – Synonymous mutations fixed in *hspColombia H. pylori* subpopulation**. Location of non – synonymous mutations with a significative Fst values in the proteins with highest number of variants positions in *hspColombia*. The 3D structure of each protein was inferred in I – TASSER server. a) HofC (HP0486). b) HopE (HP0706). c) GTP – binding protein Era (HP0517). d) Sialidase a (HP0554). e) Hypothetical protein (HP1055). f) HopF (HP0252). g) Thiol:disulphide interchange (HP0252). H) FrpB4 (HP1512). The red gradient represents the Fst value for each position.

We analysed 196,360 SNPs present in the coregenome to identify loci that have become significantly fixed in Colombian subpopulations when compared with their ancestral *hspSWEurope* subpopulation; the distribution of Fst values over the coregenome is illustrated in Figure 6. By selecting a Fst value over 0.5 we identified 26 genes significantly fixed in Colombian subpopulations, most of which encode for outer membrane proteins with a bacterial survival function (Figure 6 and Table 1). A total of 82 sites were identified in these 26 genes, 53 of these SNPs generated NS substitutions, some of these occurring in the same codon (table S4). The higher number of fixed positions and NS changes were identified in genes that encoded for the outer membrane proteins HofC (10 positions) and HopE (8 positions) as well as for Sialidase A (5 positions). In these 26 genes, there was a high number of outer membrane and transmembrane transport proteins involved in the interchange of ions and macromolecules, adhesion, bacterial adaptation to variation in the microenvironment, chemotaxis and homoeostasis [31,32]. We identified the position of each significant NS mutation in the 8 genes with the highest Fst values, that encodes the proteins HofF, Thiol:disulphide interchange protein, HofC, Era, sialidase A, HopE, an hypothetical protein, and FrpB4 (Figure 7). Additionally, to corroborate the effects of these mutations, we made an *in silico* prediction of the 3D structure of these proteins based on the consensus sequences that includes all the variants with NS changes in each subpopulation, and identified important structural changes in the HofC, HopE, Sialidase A and Thiol: disulphide interchange proteins (figure S7).

In the gene that encodes for HofC, an outer membrane protein involved in the adhesion of the bacteria to gastric epithelial cells [34,35], up to 19 fixed positions were identified, of which 10 were NS with Fst values higher than 0.94. Eight of these SNPs were concentrated in the 858–918 nucleotide positions, which correspond to the 291–309 aa residues of the protein (Figure 7a); four of these variants generate changes in acidic or alkaline aa to neutral, leading to a change in net charge (Figure 7a, figure S8a). The second gene with more changes was *hopE* that encodes for a porin involved in the adhesion to gastric epithelium, and which may induce an immune response [36]. Highly significant nonsynonymous changes were identified in the 88–208 aa residues (Figure 7b, figure S8b). Strong Fst values were also found in the GTP – binding Era protein, an essential GTPase that binds both GDP and GTP, with four significant Fst values in the 298–301 aa residues (Figure 7c, figure S8g). For the Sialidase A and the hypothetical protein HP1055, the nonsynonymous changes were also limited to a specific region of the protein (Figure 7d andE, figure S8(d,e)). We also found changes in metabolic proteins associated with protein folding, cell cycle regulation, and energy metabolism (Figure 7g andH, figure S8(g,h)).

## Discussion

Previous studies have shown the rapid and ongoing evolution of *H. pylori* populations in the Americas since 500 y ago with the encounter of European, African and Native Indigenous (Amerind) human populations [4]. These studies have found a fine adaption of *H. pylori* to the different emerging mestizo human populations, distinguishing even between neighbour countries [7,16,17]. In Colombia, a subpopulation with European ancestry but yet a high proportion of self – identity have been suggested by several groups (*hspSWEuropeColombia*) and recent reports suggest there might be more than one *H. pylori* subpopulation within Colombia [8,,13,15–17]. It has been reported that even within countries there might be different subpopulations driven by geographical separation, diet, costumes, among others. [7,7,13–17]

In Colombia, there have been important efforts to sequence the genome of a significant number of *H. pylori* isolates from different regions of the country, due to its particular epidemiological condition: a high GC mortality and a high prevalence of premalignant lesions in the central and mountainous region, and a contrasting low GC mortality in low altitude regions. The association of altitude and GC has been reported previously [10–14], but the causes for this have not yet been well elucidated. We took advantage of the significant number of genomes available from Colombian strains to perform a comprehensive study of *H. pylor*i subpopulations by analysing isolates from different regions of the country. The inclusion of many genomes from all over the world allowed us an improved resolution to identify unknown subpopulations in Colombia but also in other countries (Figure 1 and 3, table S1). Our analyses allowed us to redefine the structure of isolates from the previously named *hspSWEuropeMexico* and *hspAfrica1MiscAmerica* now defined as *hspSWEuropeNorthAmerica* and *hspSWEuropeSouthAmerica* and identified two new subpopulations composed of isolates from and Australia, *hspN Europe Australia* and *hspSWEuropeAustralia* (Figure 1).

Recent studies have documented a detailed adaptive process of *H. pylori* to different mestizo populations in Latin American countries such as Mexico, Honduras, Nicaragua, and Peru [5,7,7,15–17]. We show the presence of two subpopulations and three subgroups inside Colombia that had not been previously described and were able to identify well – differentiated clades of Colombian strains following geographical regions with a high resolution.

Our approach was to perform whole – genome analyses; whereas previous studies were limited to MLST analysis or included a reduced number of genomes [,^13^,^15–17^]. The population structure analysis revealed the existence of two clearly separated subpopulation within the Colombian isolates (Figure 1), one corresponded to the previously described *hspSWEuropeColombia* [7], grouping close to *hspSWEurope*; whereas the other was a distant cluster that we designated as *hspColombia*. Our phylogenetic analysis identified different groups, suggesting the presence of more than one evolutionary lineage, as previously suggested by Gutiérrez et al., (2017)[15] in Colombian isolates from the Cundinamarca department. Furthermore, the detailed population structure analysis indicated the presence of three subgroups within *hspColombia*, which we named after their geographic origin as *hspColombia_Nariño*, *hspColombia_Andes* and *hspColombia_Cundinamarca*, all isolates from departments in the Andes Mountains such us Nariño, Boyacá, Santander, Tolima, and Cundinamarca.

In addition to the above subpopulations, a few other Colombian strains felt into the African subpopulation *hspAfrica1NAmerica* (7), isolated from patients in the coastal region of the Nariño department that matches the African ancestry of the human population, descendent from the African slaves that established during 16th to the 19th century [37–39]. Although only two strains from Amazonian inhabitants were included in this study, they classified within the *hspIndigenous* subpopulation previously reported in native communities of Latin America [7,40]. Thus, Colombia is a complex mosaic of *H. pylori* subpopulations with European, African or Indigenous ancestry that mirror the complex array of human groups distributed along the country [8]. This study supports the existence of an evolutive differentiation process, with new subpopulations within the country that are evolving to adapt to the Colombian mestizo population from the different geographical regions.

High admixture is commonly observed in *H. pylori* genomes of European ancestry, even in the old – world regions (see Figure 5) must probably because of the constant movement of people between countries. This high admixture is also observed in genomes of the Americas with either African or European ancestry, reflecting that these populations are the result of the “recent” encounter of European, African, and Indigenous human races 500 y ago. We found that the number of SNPs reflects the differences between the *H. pylori* populations (Figure 4). However, we proved that recombination events are the main factor in the divergence of the bacterium, causing more substitutions than those caused by punctual mutations (table S3). According to this, the divergence between *H. pylori* populations might be driven mostly by substitutions generated during recombination events. Of interest, we observed that in African populations such *hspAfrica2* and *hspAfrica1SAfrica*, mutation was more frequent than recombination, suggesting that recombination is fading in the more ancient population.

Still, it is remarkable to observe that several *H. pylori* genomes of the American and Colombian subpopulations with European ancestry (*hspSWEuropeNorthAmerica*, *hspSWEuropeSouthAmerica*, *hspSWEuropeColombia* and *hspColombia*) show a reduced admixture and are mostly *painted* by their own population (self – identity ancestry), suggesting an advance process of adaption to their human host, partially explained by the presence of environmental and cultural conditions specific of each country [34–38]. Thorell et al., (2017) observed that the diversity of populations of the new world was greater than the parental population from which they evolved and suggested that this was caused by admixture after the bottlenecks produced during the drift to the Americas [17]. This “rapid” evolution may be the result of high genetic exchange whiting reproductive isolated populations where imported fragments have low diversity, homogenizing the population after few generations.

The difference between Colombian *H. pylori* subpopulations was further documented with a genomic diversity analysis that showed that, on average, *hspSWEurope* had a lower number of different SNPs than *hspSWEuropeColombia*, while the highest number of SNPs were observed when the comparison was made against *hspColombia* (Figure 4). Of note, we observed that recombination is   12 (10.554–13.914) times more important than mutation in the divergence of these populations, showing that recombination plays a major role in the differentiation – evolution of these populations. Interestingly, this effect varies between strains with high self – identity and those with high admixture within each population, resulting markedly lower for self – identity strains, with a factor of 11 times more important for mixed strains in the *hspSWEuropeColombia* subpopulation and 6 times for mixed strains of *hspColombia*. On the other hand, the recombination effect was 6 times more important in self – identity strains of *hspColombia* than in strains of *hspSWEuropeColombia*. This might be explained by frequent events of recombination in a population that was quickly isolated after the bottleneck produced during the Spanish expansion to the Americas. Alternatively, this may have resulted due to a selective pressure in the gastric niche that favoured the stochastic elimination of lineages and the homogenization of alleles, leading to a reduced allelic diversity available for further recombination events.

We observed the same major role of recombination in other populations in the Americas, such as *hspSWEuropeNorthAmerica* and *hspSWEuropeSouthAmerica*, that may be explained as in the Colombian populations, since they were derived from the same parental population (*hspSWEurope*) that arrived at the New World at similar times during the conquest. This proves that recombination in *H. pylori* has resulted in well – differentiated populations in a relatively short period of time. In addition, strains with self – identity were observed in other world – wide populations, such as *hpAfrica2*, *hspEastAsia*, *hspIndigenous*, *hpSahul*, *hspAfrica1SAfrica* and *hspNEuropeAustralia*, which are ancient populations where isolation may also have favoured the homogenization of the allele frequencies.

## Membrane proteins drive the differentiation of Colombian *H.* *pylori* subpopulation

The Fst analysis allowed the identification of the genes that contributed the most to the differentiation of the Colombian subpopulations and identified genes that encoded for proteins involved in bacterial survival and central metabolism. A large number of SNP positions were identified in membrane proteins associated with transport, structure, adhesion, and homoeostasis, which play an essential role in the interaction and adaptation to the host [34,36]. These results suggest that the changes observed in host – interaction proteins play a major role in differentiation of *H. pylori* populations in Colombia, which is in agreement with recent reports in other Latin American subpopulations [7]. In fact, 10 of the proteins with significant Fst values concur in both studies, including the outer membrane proteins *hofC*, *hopC*, the transport protein *fecA* and the isomerase *ppiC*. Still, there were genes like *frpB4* and *hopF* with high Fst values for Colombian populations that were not reported in the Latin American study [7], suggesting the existence of specific adaptions for some populations.

To estimate the impact of the SNPs identified with significant Fst values, we analysed the protein sequence and predicted the 3D structure of the seven genes with highest Fst values and estimated the consensus sequence including all the variants with NS changes by subpopulation. Crystallography data was available for only two proteins, while for the remaining proteins the 3D structures had to be predicted with an *in – silico* model based on the consensus sequence of its parental subpopulation *hspSWEurope*, with the limitations that these predictive models present (Figure 7 and figure S7). Still, the structural changes identified in the HofC, HopE, Sialidase A and Thiol: disulphide interchange membrane proteins of the *hspColombia* strains (figure S7) show significant structural changes in these proteins, which most probably are associated with the adaptation process of *H. pylori* to the gastric epithelium of Colombian people, particularly to the host molecules these proteins interact with. However, more studies are required to corroborate and estimate the impact of these changes in the function and interactions of the proteins, including their crystallography 3D structures.

The *hofC* gene presented the largest number of positions with significant Fst values, most of them localized in the 858–918 nucleotide region, which agrees with the report by Thorell et al., (2017) [17] that also found several SNPs in *hofC* in other Latin American *H. pylori* subpopulations. Many of the identified SNPs caused amino acid changes modifying polarity, charge or pH, which as we showed may modify substantially the structure of the protein and its function (figure S7a).

The outer membrane proteins HofC and HopC may function as adhesins recognizing receptors on the surface of the gastric epithelial cells and both, the human receptor and the *H. pylori* adhesins may have co – evolved to have the right interaction. This is less clear for FecA, that transport ferric citrate or for the isomerase PpiC; probably they also interact with human proteins. We also found changes in proteins that regulate concentration of iron and potassium. The causal mechanisms underlying this association are not clear, but it probably reflects the response of *H. pylori* to the high concentrations of ions in the water and food due to Colombian geology and diet, especially in the mountain areas of the country [41]. The interaction of these proteins with proteins or other molecules of the host in the environment of the gastric mucosa exert a strong positive selection to attain the right adaption of the bacterial – host interaction. In genes like those coding for outer membrane proteins HofC and HopE, fixed SNPs occur in more than one position (Table 1 and Figure 7) suggesting that in some genes mutations in multiple sites are needed to get the right changes in the protein to adapt to the stomach niche of the regional human population. In this study, the outer membrane protein genes *hofC*, *hopE* and *frpB4* had the highest Fst values identified, indicating that they play a major role in the adaptation of *H. pylori* to the Colombian human population, and are under strong selective pressure to drive the evolutive process during the differentiation of the populations.

In conclusion, the findings described in this study demonstrate that *H. pylori* have evolved in Colombia to give rise to new subpopulations that follow a geographic structure. The evidence suggests that this evolutive process is in progress, with some strains having already a genome with almost 100% self – identity, but others with gradients of admixture. The study also show that outer membrane protein genes present the highest fixation values and that high recombination rates seem to drive the evolutive process as a result of high selective pressure by the host.

## Supplementary Material

Supplemental Material

## Data Availability

The *H. pylori* genomes that support the findings of this study are publicly available at Enterobase (https://doi.org/10.1080/21505594.2022.2095737), Genbank (https://doi.org/10.1080/21505594.2022.2095737), and BIGSdb (https://doi.org/10.1080/21505594.2022.2095737).

## Data deposition

The information into this paper was deposited in Biorxiv and is available at: https://biorxiv.org/cgi/content/short/2021.12.14.472690v1.
